# Exploring the common diagnostic gene KCNJ15 and shared pathway of ankylosing spondylitis and ulcerative colitis through integrated bioinformatics

**DOI:** 10.3389/fphys.2023.1146538

**Published:** 2023-05-05

**Authors:** Su-Zhe Zhou, Li Shen, Zhong-Biao Fu, Hao Li, Ying-Lian Pan, Run-Ze Yu

**Affiliations:** ^1^ Department of Orthopedics, Anhui No 2 Provincial People’s Hospital, Hefei, China; ^2^ Department of General Practice, Hefei BOE Hospital, Hefei, China; ^3^ Beijing United Family Hospital, Beijing, China; ^4^ Department of Gastroenterology, The Gastroenterology Clinical Medical Center of Hainan Province, The Second Affiliated Hospital of Hai Nan Medical University, Haikou, China; ^5^ Graduate School, Tianjin Medical University, Tianjin, China; ^6^ Department of Medical Oncology, The First Affiliated Hospital of Hainan Medical University, Haikou, China

**Keywords:** ankylosing spondylitis, ulcerative colitis, WGCNA, machine learning algorithm, immune cells infiltration

## Abstract

**Introduction:** The similarity between ankylosing spondylitis (AS) and ulcerative colitis (UC) in incidence rate and pathogenesis has been revealed. But the common pathogenesis that explains the relationship between AS and UC is still lacked, and the related genetic research is limited. We purposed to explore shared biomarkers and pathways of AS and UC through integrated bioinformatics.

**Methods:** Gene expression data of AS and UC were obtained in the GEO database. We applied weighted gene co-expression network analysis (WGCNA) to identify AS-related and UC-related co-expression gene modules. Subsequently, machine learning algorithm was used to further screen hub genes. We validated the expression level and diagnostic efficiency of the shared diagnostic gene of AS and UC in external datasets. Gene set enrichment analysis (GSEA) was applied to analyze pathway-level changes between disease group and normal group. Finally, we analyzed the relationship between hub biomarker and immune microenvironment by using the CIBERSORT deconvolution algorithm.

**Results:** 203 genes were obtained by overlapping AS-related gene module and UC-related gene module. Through SVM-RFE algorithm, 19 hub diagnostic genes were selected for AS in GSE25101 and 6 hub diagnostic genes were selected for UC in GSE94648. KCNJ15 was obtained as a common diagnostic gene of AS and UC. The expression of KCNJ15 was validated in independent datasets, and the results showed that KCNJ15 were similarly upregulated in AS samples and UC samples. Besides, ROC analysis also revealed that KCNJ15 had good diagnostic efficacy. The GSEA analysis revealed that oxidative phosphorylation pathway was the shared pathway of AS and UC. In addition, CIBERSORT results revealed the correlation between KCNJ15 gene and immune microenvironment in AS and UC.

**Conclusion:** We have explored a common diagnostic gene KCNJ15 and a shared oxidative phosphorylation pathway of AS and UC through integrated bioinformatics, which may provide a potential diagnostic biomarker and novel insight for studying the mechanism of AS-related UC.

## 1 Introduction

Spondyloarthritis (SpA) is a set of autoimmune-related chronic inflammatory rheumatic diseases, including ankylosing spondylitis (AS), inflammatory bowel disease (IBD), undifferentiated spinal arthritis and juvenile chronic arthritis (Sharip and Kunz, 2020). AS is the prototype of SpA, which mainly involves the spine and sacroiliac joints, and is characterized by pain and stiffness in the lower back or buttocks (Ritchlin and Adamopoulos, 2021). IBD is characterized by abdominal pain, diarrhea, bloody stool and weight loss (Stolwijk, Essers, et al., 2015; Fragoulis et al., 2019).

IBD is one of the most common extra-articular manifestations of AS (Fragoulis et al., 2019; Sharip and Kunz, 2020). The prevalence of clinically evident IBD in AS patients is about 6%–14% (Essers et al., 2015; de Winter et al., 2016). Besides, studies have reported that more than 50% of AS patients have occult subclinical intestinal inflammation (Van Praet et al., 2013; Stolwijk, van Tubergen, et al., 2015; Cypers et al., 2016). Meanwhile, studies revealed that intestinal inflammation had an important impact on the pathogenesis of AS, which may not only progress to more severe IBD manifestations, but also have a certain impact on the prognosis of arthritis (Rogler et al., 2021). It is estimated that about 2%–16% of IBD patients have combined with AS (Stolwijk et al., 2013). In addition, the expertise of the therapist determines the clinical and laboratory tools used for disease assessment, which in turn guides treatment decisions that may ignore the affected system or even in the opposite direction (Gionchetti and Rizzello, 2016). Increasing awareness of intestinal and musculoskeletal manifestations among rheumatologists and gastroenterologists will lead to early diagnosis and multidisciplinary approaches, especially in the field of pharmacologic therapy.

However, the common pathogenic mechanism that explains the relationship between IBD and AS is still unclear (Zioga et al., 2022), and the related genetic research is also limited. The purpose of this study is to explore shared biomarkers and molecular mechanisms of AS and UC. We have analyzed mRNA expression data published on Gene Expression Omnibus (GEO). Finally, KCNJ15 was obtained as a common diagnostic biomarker of AS and UC by combining weighted gene co-expression network analysis (WGCNA) and machine learning algorithm. Besides, Gene set enrichment analysis (GSEA) results revealed that oxidative phosphorylation pathway may associated with intestinal involvement in AS. CIBERSORT results revealed that KCNJ15 participated in the changes of immune microenvironment in AS and UC. In words, we have explored a common diagnostic gene KCNJ15 and a shared oxidative phosphorylation pathway AS and UC, which provided a potential diagnostic biomarker and novel insight for studying the mechanism of AS-related UC.

## 2 Materials and methods

### 2.1 Gene expression profile data

We searched RNA-seq profiles of AS and UC published in GEO database. The number of samples in the normal group and the disease group should more than 15. Finally, the microarray datasets of AS (GSE25101 and GSE73754) and UC (GSE94648 and GSE38713) were downloaded. Details of the above datasets showed in Table 1. Among them, GSE73754 and GSE38713 were selected as independent validation set for AS and UC respectively.

**TABLE 1:** Details of the datasets.

Datasets	Disease	Study samples	Control samples
GSE25101	AS	16	16
GSE94648	UC	17	22
GSE73754	AS	52	20
GSE38713	UC	15	13

### 2.2 Weighted gene co-expression network analysis

“WGCNA” R package was used to construct weighted gene co-expression networks and identify gene modules related to AS and UC (Langfelder and Horvath, 2008). We used the “pickSoftThreshold” function to analyze the scale independence and average connectivity of modules under different power values, and determine the optimal soft threshold *β*. We calculated co-expression similarity and adjacency, and further constructed topological overlay matrix (TOM). We used the hierarchical clustering function to classify genes with similar expression into the same module. The minimum gene number of modules was 50, and the module merging threshold was 0.25. The dynamic tree was used to cut and identify co-expression modules, and then the modules with similar expression patterns were merged. Then, we evaluate the correlation between modules and clinical characteristic by Pearson correlation analysis. *p* < 0.05 were considered statistically significant. We identified the module with highest correlation coefficient as AS-related and UC-related module. The online Venn diagram tool was used to obtain their potential common genes by overlapping the AS-related and UC-related modules.

### 2.3 Identification and validation of diagnostic biomarkers

As an effective characteristic selection algorithm, SVM-RFE has been widely used to select hub diagnostic genes (Lin et al., 2012). We applied SVM-RFE to further select core diagnostic genes from the above intersected genes of the AS-related module and UC-related gene module by using the “e1071” R package (Yoon and Kim, 2009). We calculated the area under the ROC curve (AUC) to evaluate the diagnostic performance of core diagnostic genes by using the “pROC” R package.

### 2.4 Gene set enrichment analysis

We performed GSEA to analyze pathway-level changes between disease group and normal group using “ClusterProfiler” R package. GSEA annotated gene set (c2. Cp. Kegg. v7.5.1. symbols. gmt) was downloaded from MSigDB database (Liberzon et al., 2015). The enrichment KEGG pathways with *p* < 0.05 were considered statistically significant. The results were visualized using “enrichplot” package in R software.

### 2.5 Immune analysis algorithm

For performing immune cells infiltration analysis, we applied the CIBERSORT deconvolution algorithm to analysis the proportion of 22 types of immune cells in AS samples (GSE73754) and UC samples (GSE38713) on the basic of immune cell-related genes expression levels (Chen et al., 2018). The “vioplot” R package was applied to study the infiltration level of immune cells between different groups. The correlation between hub gene and immune cells infiltration level was explored by Spearman correlation analysis.

## 3 Results

### 3.1 Construction of weighted gene co-expression networks

**FIGURE 1:**
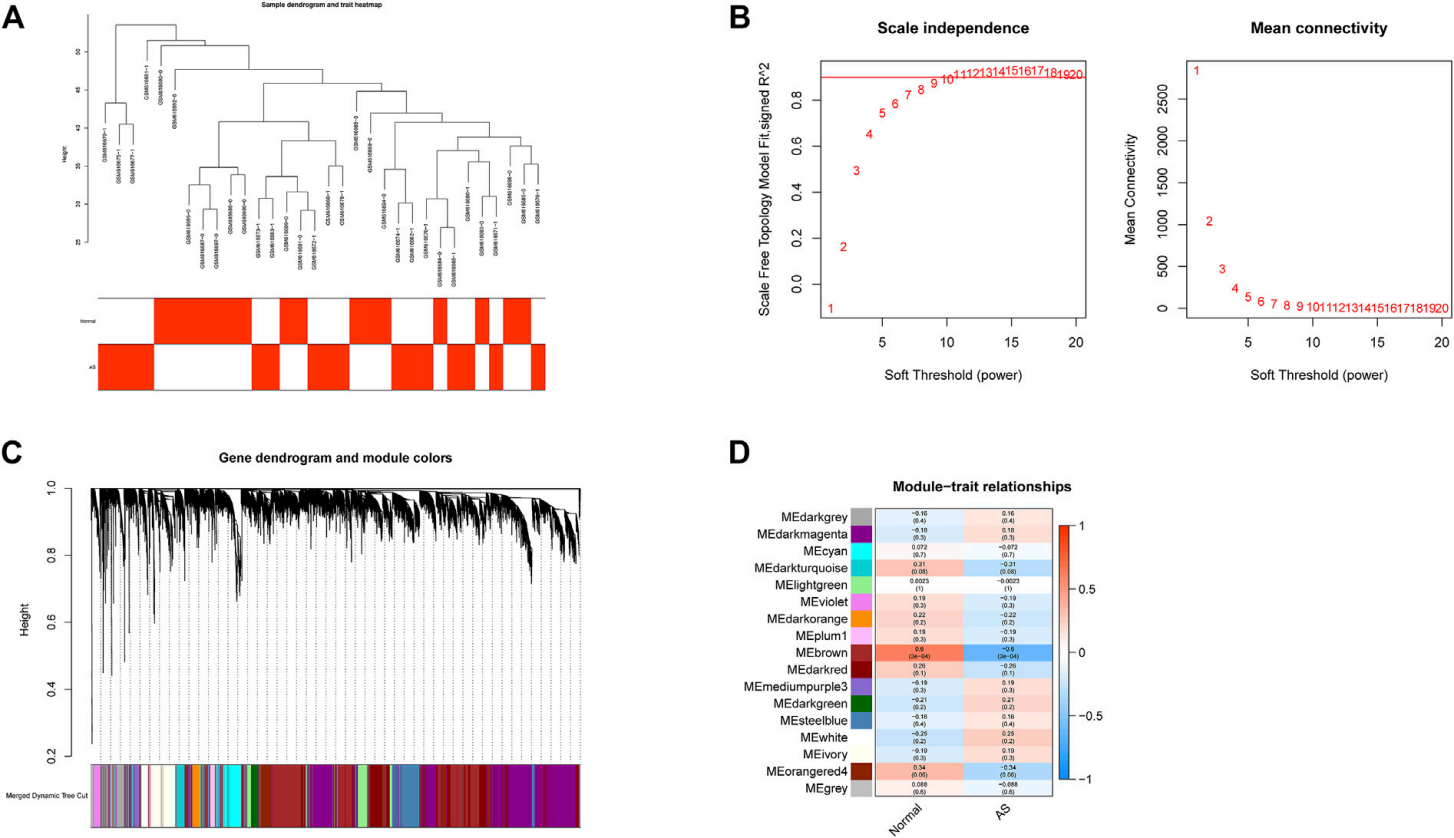
Construction of weighted gene co-expression networks in GSE25101. **(A)** Clustering dendrogram of samples. **(B)** Network topology analysis of different soft threshold power. **(C)** Dendrogram of the gene modules. **(D)** The correlation coefficients between gene modules and AS occurrence.

**FIGURE 2:**
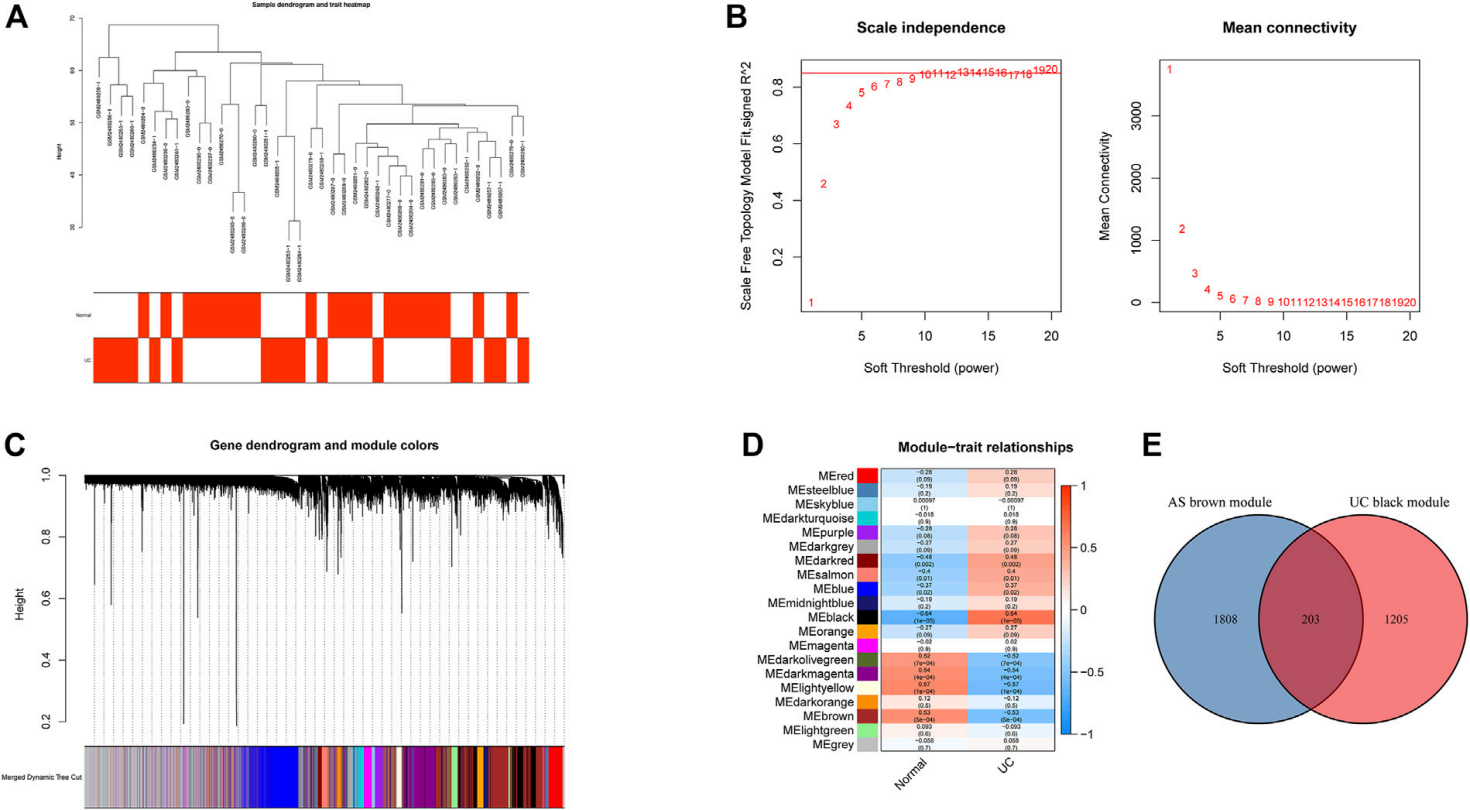
Construction of weighted gene co-expression networks in GSE94648. **(A)** Clustering dendrogram of samples. **(B)** Network topology analysis of different soft threshold power. **(C)** Dendrogram of the gene modules. **(D)** The correlation coefficients between gene modules and UC occurrence. **(E)** Venn diagram of AS-related genes and UC-related genes.

All samples in GSE25101 and GSE94648 were clustered and included in the subsequent analysis **(** Figure 1A; 2A). The optimal soft threshold power *β* was 9 for GSE25101 with scale-free *R* ^2^ = 0.9 (Figure 1B). A total of 17 modules were identified after merging modules with similar expression patterns using dynamic tree cutting (Figure 1C). In GSE94648, as the soft threshold power *β* = 13 with scale-free *R* ^2^ = 0.85, the connectivity between genes follows a scale-free network distribution (Figure 2B). According to the TOM matrix, a hierarchical clustering tree was constructed among genes, and the total number of gene modules after merging was 20 using dynamic tree cutting. Each module has a unique color as an identifier (Figure 2C). Then, the correlations between clinical characteristic and modules were calculated. The brown module had the strongest relationship with AS (correlation coefficient = 0.6) in GSE25101 (Figure 1D). The black module had the strongest relationship with UC (correlation coefficient = 0.64) in GSE94648 (Figure 2D). Then, 203 common genes were obtained by overlapping AS-related module and UC-related module (Figure 2E).

### 3.2 Identification of common diagnostic gene by machine learning algorithm

**FIGURE 3:**
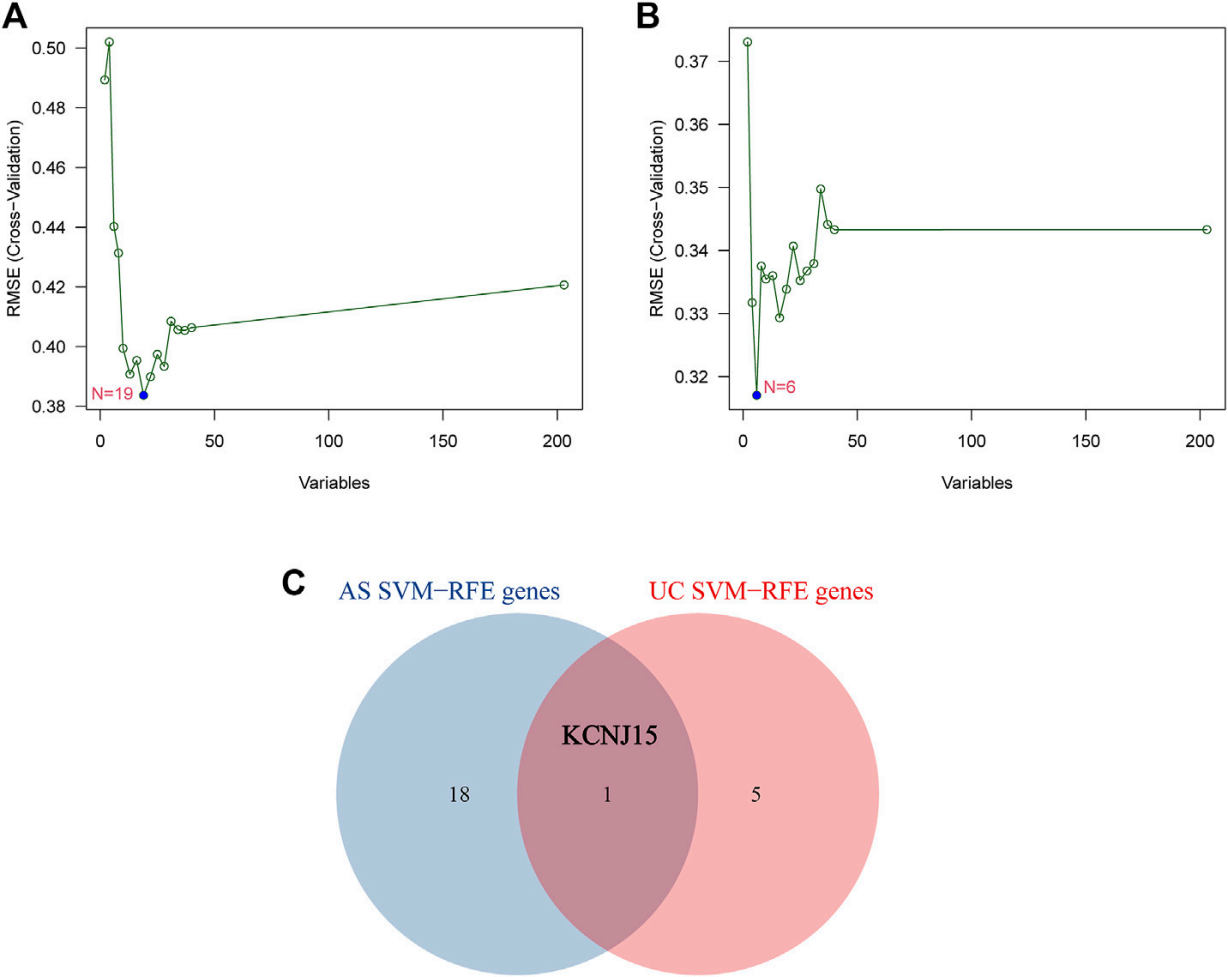
Identification of diagnostic genes using SVM-RFE algorithm. **(A)** Feature genes selection in GSE25101. **(B)** Feature genes selection in GSE94648. **(C)** Venn diagram of overlapping feature genes.

SVM-RFE was performed to further screen diagnostic genes based on the above 203 intersected genes of the AS-related module and UC-related module. The SVM-RFE analysis results show that 19 candidate diagnostic genes (NEDD8; DDIT3; RHOA; TXN; TNIP2; COP1; GTPBP3; TRIM32; HOXC4; LSM10; CLEC3B; HTATIP2; BCOR; VPS25; SART3; KCNJ15; HSBP1; TSPAN18; SCCPDH) were screened out in GSE25101 and 6 candidate diagnostic genes (SLC22A4; ANKRD22; MAPK14; MR1; KCNJ15; RABGAP1) were screened in GSE94648 (Figures 3A,B). Finally, KCNJ15 was identified as the common diagnostic gene of AS and UC (Figure 3C).

### 3.3 Validation of hub diagnostic genes

**FIGURE 4:**
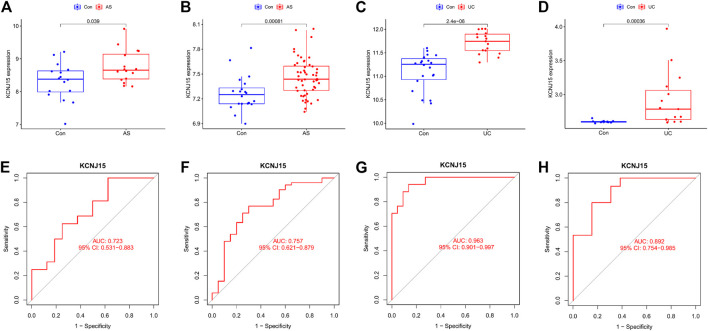
Validation of the expression level and diagnostic efficacy of KCNJ15 gene. The box plots of KCNJ15 gene in GSE25101 **(A)**, GSE73754 **(B)**, GSE94648 **(C)** and GSE38713 **(D)**. The ROC curves of KCNJ15 gene in GSE25101 **(E)**, GSE73754 **(F)**, GSE94648**(G)** and GSE38713 **(H)**.

We validated the expression level of KCNJ15 in GSE25101, GSE94648 and external independent datasets (GSE73754 and GSE38713). KCNJ15 was similarly upregulated in AS and UC samples (Figures 4A–D). In addition, the ROC analysis results showed that KCNJ15 had good diagnostic efficacy in GSE2510 (AUC = 0.723) and GSE94648 (AUC = 0.963) (Figures 4E, F). In the external independent datasets, KCNJ15 also showed potent diagnostic efficacy in GSE73754 (AUC = 0.757) and GSE38713 (AUC = 0.892) (Figures 4G, H).

### 3.4 Gene set enrichment analysis

**FIGURE 5:**
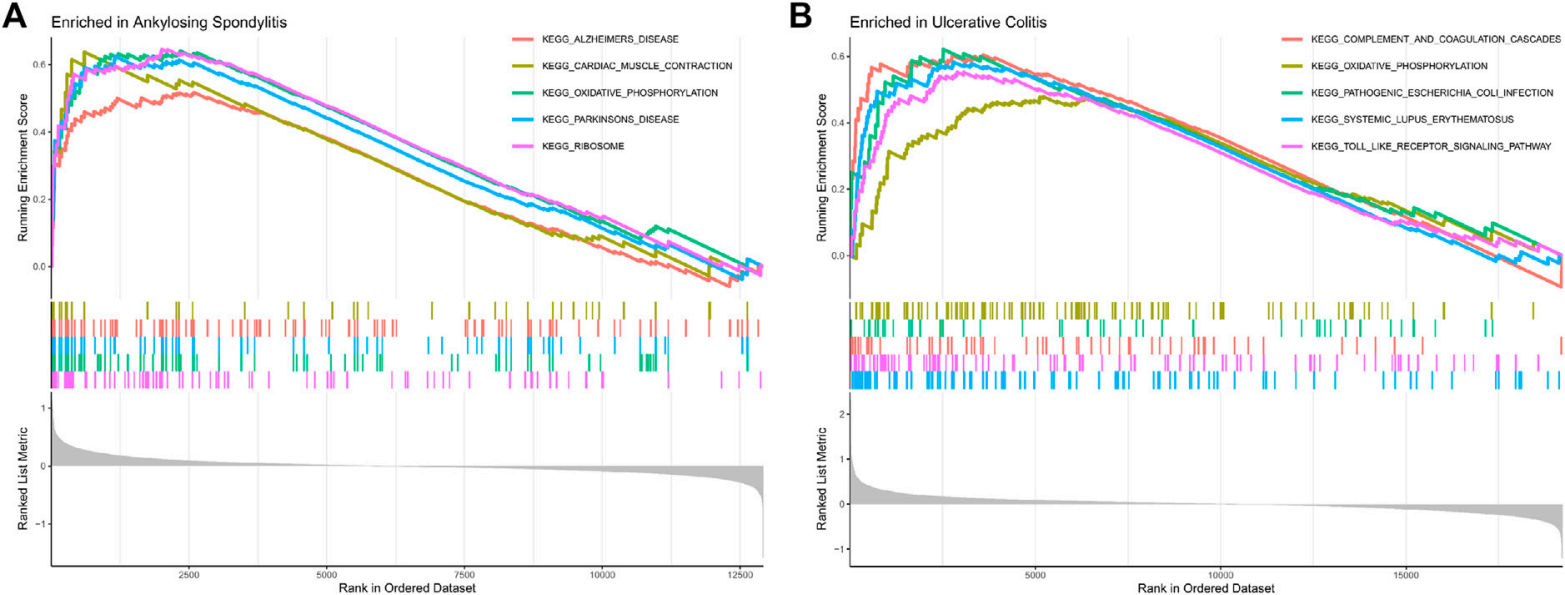
**(A)** GSEA analyses results of AS samples. **(B)** GSEA analyses results of UC samples.

The GSEA results showed that the oxidative phosphorylation and ribosomal pathway were positive enrichment in AS samples (Figure 5A), and the oxidative phosphorylation, pathogenic *Escherichia coli* infection, systemic lupus erythematosus and Toll-like receptor pathway were positive enrichment in UC samples (Figure 5B). Oxidative phosphorylation pathway was the shared enrichment signaling pathway of AS and UC.

### 3.5 Immune cells infiltration analysis

**FIGURE 6:**
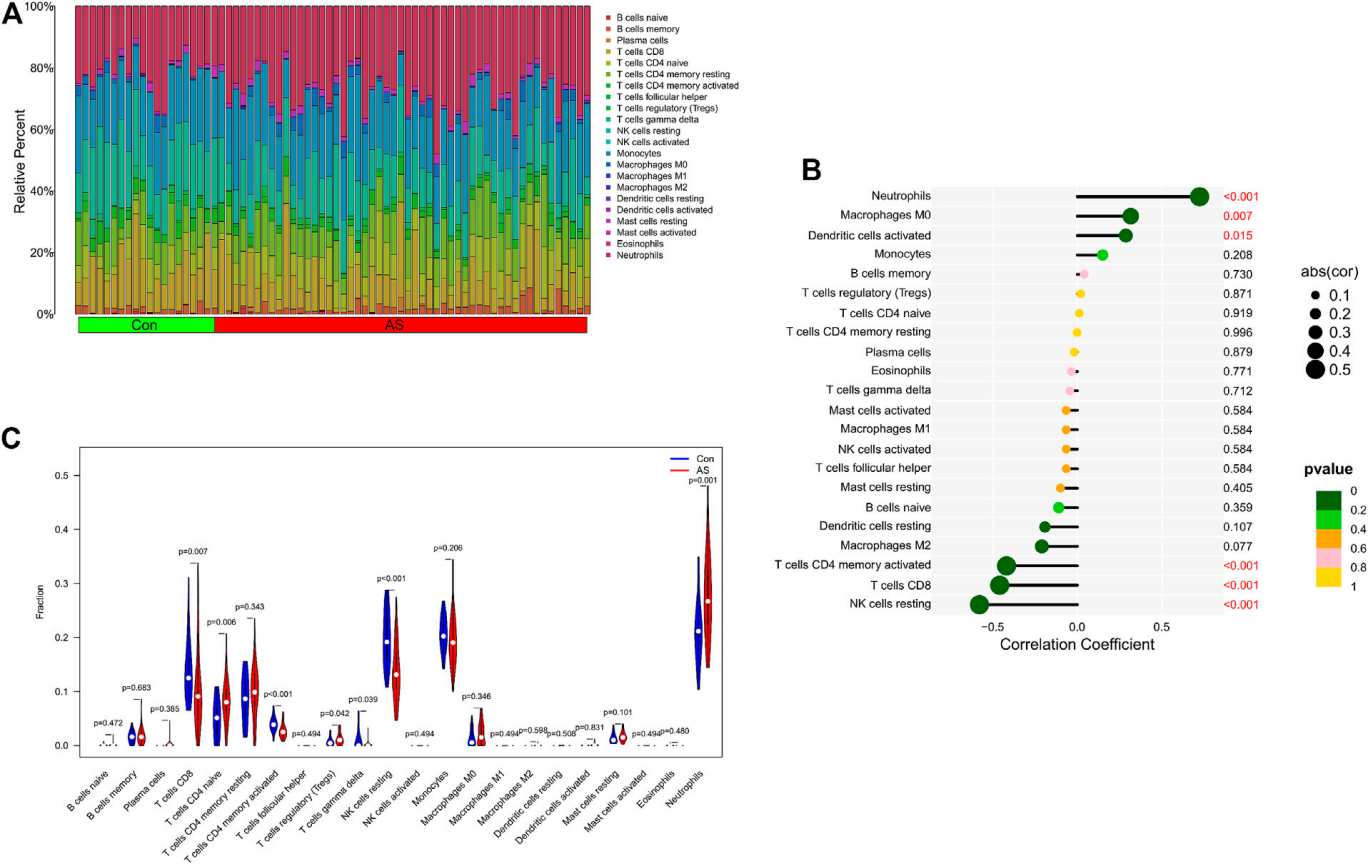
Immune infiltration analysis of KCNJ15 gene in AS. **(A)** Histogram of proportion of immune cells. **(B)** Correlation between KCNJ15 and immune cells content. **(C)** Proportion of 22 kinds of immune cells in a violin diagram.

**FIGURE 7:**
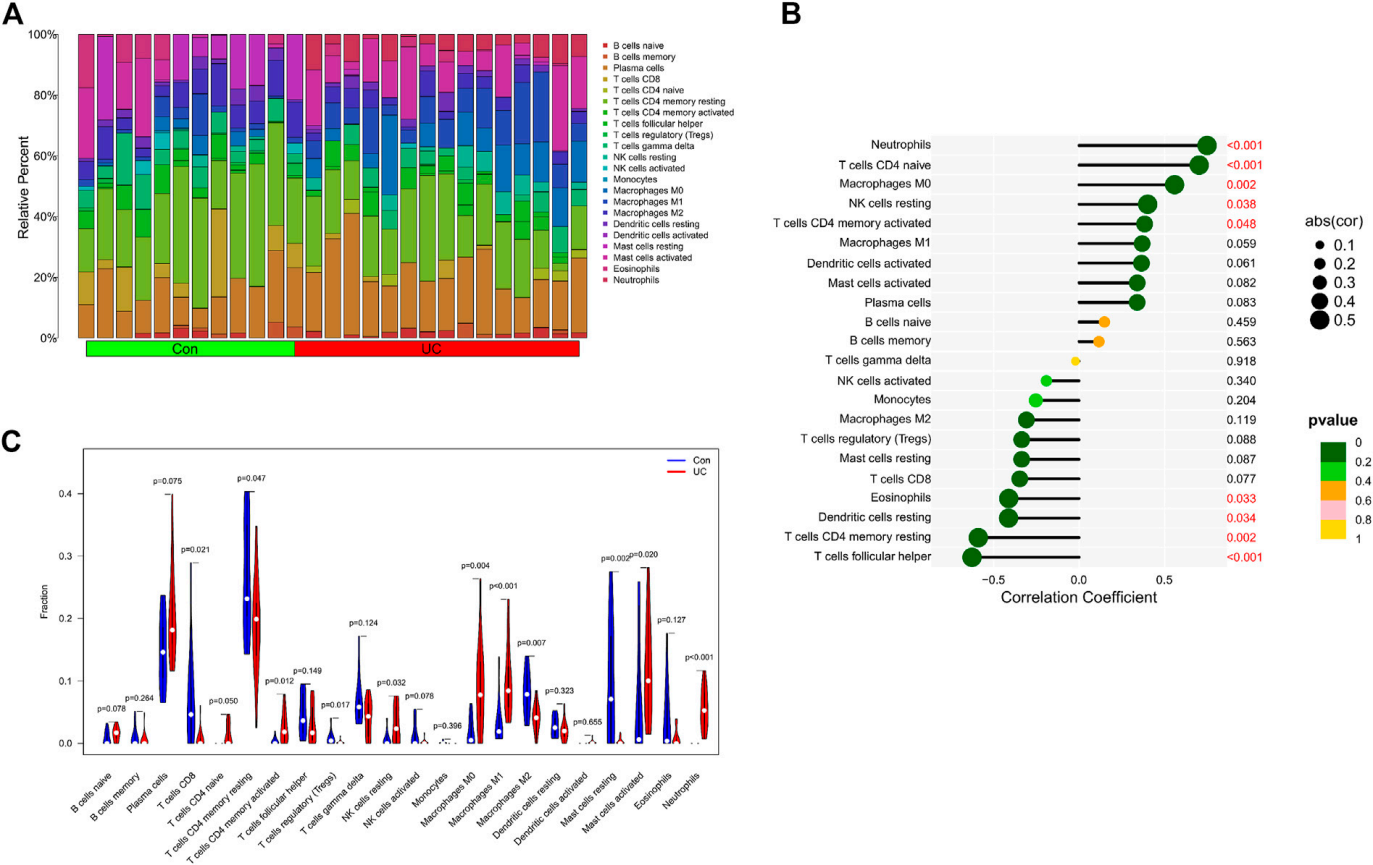
Immune infiltration analysis of KCNJ15 gene in UC. **(A)** Histogram of proportion of immune cells. **(B)** Correlation between KCNJ15 and immune cells content. **(C)** Proportion of 22 kinds of immune cells in a violin diagram.

We further explored the relationship between KCNJ15 gene and immune microenvironment. We analyzed the percentages of 22 kinds of immune cells in AS and UC samples (Figure 6A; 7A). The ratio of Neutrophils in AS samples was higher. However, the ratios of resting NK cells, CD8 (+) T cells and activated memory CD4 (+) T cells in AS samples were lower (Figure 6C). The ratios of Neutrophils, M0 and M1 macrophages in UC samples were markedly higher compared to normal control samples, and the ratio of resting memory CD4 (+) T cells in UC samples was lower than that normal control samples (Figure 7C). Moreover, we analyzed the correlation between KCNJ15 gene and immune cells content. KCNJ15 was positively correlated with Neutrophils cells content (R = 0.72), and negatively correlated with resting NK cells (R = −0.58) and CD8 (+) T cells (R = −0.46) in AS (Figure 6B). In UC, KCNJ15 had a positive relation to Neutrophils cells content (R = 0.75) and a negative relation to follicular helper T cells content (R = −0.63) (Figure 7B). The above results were statistically significant (*p* < 0.05), revealing the relationship between KCNJ15 and immune activity.

## 4 Discussion

AS is a chronic inflammatory disease, mainly involving the spine and sacroiliac joint. It is the most common subtype of SpA (Garcia-Montoya, Gul, and Emery, 2018). IBD is a group of chronic inflammatory diseases involving the gastrointestinal tract, including UC and CD. Studies have revealed the similarity between AS and IBD in incidence rate and pathogenesis (Gracey et al., 2020). There is overlap in the drugs used to treat AS and IBD, although the main symptoms of AS and IBD are low back pain and intestinal symptoms respectively (Feagan et al., 2016).

The comorbidity of AS and IBD brings great challenges to the doctors in the department of digestion and rheumatism. AS and IBD are usually managed as separate diseases, which has a profound impact on diagnosis and treatment. In one survey, more than 30% of patients with IBD as the first symptom had SpA related symptoms, but 50% of patients had never been to the rheumatic department. Researchers found that insufficient diagnosis or delayed treatment of these patients often led to disease progression and decreased quality of life. With the wide application of IL-17 inhibitors in patients with AS, people are increasingly worried that IL-17 inhibitors may aggravate intestinal inflammation in IBD patients (Penso et al., 2022). Therefore, it is urgent to clarify the disease mechanism between the two. At present, the etiological mechanism of AS-related UC is still unclear.

The completion of the Human Genome Project in 2003 marks that human beings have stepped into the era of bio-information technology. In the following decades, mining massive genetic data has become a research hotspot. Among them, the application of microarray and high-throughput gene expression profiling technology to analyze the transcriptome of diseases has been widely spread in the research of chronic inflammatory diseases. We reanalyzed the RNA-seq profiles of AS and UC obtained from the GEO database. Ultimately, a common diagnostic gene KCNJ15 and a shared oxidative phosphorylation pathway of AS and UC were explored through integrated bioinformatics. We further validated that KCNJ15 had potent value as diagnostic biomarker for UC and AS.

Both AS and UC are immune-mediated chronic inflammatory diseases (Du and Ha, 2020; Voruganti and Bowness, 2020; Mauro et al., 2021). In this study, we have explored the role of immune cell infiltration in the pathogenesis of AS and UC. “CIBERSORT” algorithm was applied to estimate the composition of immune cells. The results showed that compared with the normal group, Neutrophils cells were increased in AS, while resting NK cells, CD8 (+) T cells and activated memory CD4 (+) were decreased. Neutrophils cells, M0 and M1 Macrophages cells in UC samples were increased in UC samples, while resting memory CD4 (+) T cells in UC samples were decreased. In addition, the correlation analysis results showed that KCNJ15 was significantly correlated with the above immune cells with different immune infiltration levels.

Oxidative phosphorylation plays an important role in many cancer subtypes (Sica et al., 2020). It not only provides energy for tumor cell growth, but also promotes the occurrence and development of various tumors. Oxidative phosphorylation is a key process that connects the tricarboxylic acid cycle with adenosine triphosphate (ATP) production, and it is also the final biochemical pathway of ATP production (Papa et al., 2012; Nolfi-Donegan, Braganza, and Shiva, 2020). In our study, oxidative phosphorylation pathway was the shared enrichment signaling pathway of AS and UC samples. Studies have shown that metformin can inhibit oxidative phosphorylation (He, 2020). In a long-term follow-up study, a significant reduction in the incidence rate of IBD was found in T2DM patients treated with metformin (Tseng, 2021). Besides, metformin also has therapeutic effect on autoimmune inflammatory rheumatic diseases, such as osteoarthritis (OA), rheumatoid arthritis (RA) and AS (Lee et al., 2015; Zheng et al., 2021; Kim, Choe, and Park, 2022). Therefore, we speculated that metformin may slow down ankylosing spondylitis and ulcerative colitis by reducing oxidative phosphorylation.

There are also some limitations in this study. First of all, the function of hub gene KCNJ15 needs to be further verified *in vitro* and *in vivo*, which will be the focus of our future work. Secondly, the sample size in the expression profile datasets used in the study is small. More samples are needed to further verify the diagnostic value of key genes in the future.

## 5 Conclusion

This study has identified KCNJ15 gene as a common diagnostic gene of AS and UC by combining WGCNA and machine learning algorithm. We further validated the diagnostic efficacy of KCNJ15 gene in the independent datasets. Moreover, CIBERSORT results revealed the relationship between KCNJ15 and immune cells infiltration. GSEA results revealed that oxidative phosphorylation pathway was the shared enrichment pathway of AS and UC samples. This study provided a potential diagnostic biomarker and novel insight for studying the mechanism of AS-related UC.

## Data Availability

The datasets presented in this study can be found in online repositories. The names of the repository/repositories and accession number(s) can be found in the article/supplementary material.
